# The Efficacy of Prone Single-Position Lateral Lumbar Interbody Fusion for Symptomatic Cranial Adjacent Segment Degeneration

**DOI:** 10.3390/jcm15020895

**Published:** 2026-01-22

**Authors:** Dong Hun Kim, Sang Don Kim, Jung-Woo Hur, Jin Young Kim, Jae Taek Hong

**Affiliations:** 1 Bucheon St. Mary’s Hospital, The Catholic University of Korea, Seoul 06591, Republic of Korea; 21400254@cmcnu.or.kr (D.H.K.); 10503978@cmcnu.or.kr (S.D.K.); 2 Eunpyeong St. Mary’s Hospital, The Catholic University of Korea, Seoul 06591, Republic of Korea; 22400111@cmcnu.or.kr (J.Y.K.);

**Keywords:** prone single-position surgery, lateral lumbar interbody fusion, adjacent segment degeneration, transpsoas approach

## Abstract

**Background/Objectives**: Following lumbar fusion procedures, adjacent segment degeneration (ASD) at cranial levels presents as a well-documented long-term complication, manifesting through recurrent pain, neurological deficits, and progressive functional decline. The prone single-position technique for lateral lumbar interbody fusion (PSP-LLIF) streamlines surgical workflow by eliminating the need for intraoperative patient repositioning; however, comprehensive evidence supporting its clinical and radiological effectiveness in managing cranial ASD remains insufficient. **Material and Methods**: This retrospective cohort study examined 30 consecutive patients presenting with symptomatic cranial adjacent segment disease who were treated with PSP-LLIF at a single institution. Patient-reported outcome measures included visual analog scale (VAS) assessments for axial and radicular pain, alongside the Oswestry Disability Index (ODI) for functional status evaluation. Radiological parameters included overall and segmental lumbar lordotic measurements, anterior and posterior disk height, fusion status, and instrumentation-related complications. **Results:** At 12-month postoperative evaluation, substantial clinical improvements were demonstrated. Mean VAS reductions measured 4.7 points for axial pain and 6.5 points for radicular pain, while ODI decreased by 28.5 points (*p* < 0.05). Radiological assessment demonstrated mean increases of 6.3° in lumbar lordosis and 5.1° in segmental lordosis, along with significant gains in both anterior and posterior disk height (*p* < 0.05). Solid fusion was radiographically confirmed at all instrumented levels. Temporary postoperative neurological symptoms developed in several patients but resolved spontaneously without requiring revision surgery. **Conclusions:** PSP-LLIF yields substantial clinical benefit and reliable radiological correction in patients with symptomatic cranial ASD. Optimal outcomes necessitate rigorous adherence to position-specific technical modifications, particularly maintenance of perpendicular fluoroscopic trajectories and implementation of continuous neural monitoring to account for prone-induced anatomical shifts. This approach represents a viable treatment strategy for patients with symptomatic cranial ASD.

## 1. Introduction

Adjacent segment degeneration (ASD) remains one of the most challenging long-term complications following lumbar spinal fusion. Symptomatic cases occur in approximately 10–20% of patients and often require revision surgery [1,2,3]. Traditional revision through a posterior approach carries significant risks, with dural tear rates ranging from 13.2% to 15.9%, substantially higher than the 3.5% to 7.6% observed in primary posterior surgeries [4,5]. Revision surgery necessitated by ASD inevitably involves re-exposure of previously dissected paraspinal muscles, leading to additional muscle injury that may subsequently cause increased postoperative pain and decreased functional capacity [6].

Lateral lumbar interbody fusion has emerged as an alternative that avoids these complications. Recent studies have demonstrated that oblique lumbar interbody fusion (OLIF) for adjacent segment disease achieves better outcomes than revision posterior surgery, with shorter operative times, reduced blood loss, and lower dural tear rates [7,8,9]. However, conventional lateral approaches require the patient to be positioned in lateral decubitus for the interbody fusion, followed by repositioning to the prone position for posterior instrumentation. This process is time-consuming and involves multiple steps, including coordination with anesthesia staff, patient repositioning, bed changes, and re-draping.

To address this limitation, surgeons developed a technique to perform lateral interbody fusion entirely in the prone position, eliminating the need for intraoperative repositioning. This prone single-position lateral lumbar interbody fusion (PSP-LLIF) technique maintains the advantages of lateral access while allowing for posterior instrumentation to be performed in the same position [10,11]. While a recent multicenter study has demonstrated the feasibility and efficiency of PSP-LLIF for revision lumbar fusion, the included cohorts were heterogeneous in terms of revision indications, operated levels, and surgical complexity [10]. Therefore, the present study aimed to evaluate the clinical and radiological outcomes of PSP-LLIF specifically in patients undergoing revision surgery for symptomatic cranial ASD, and to describe the technical considerations necessary for its safe and effective application.

## 2. Materials and Methods

### 2.1. Patient Selection

This retrospective study was approved by the Institutional Review Board of The Catholic University of Korea. We conducted a retrospective review of 30 consecutive patients with symptomatic cranial adjacent segment degeneration who underwent PSP-LLIF at a single institution between June 2023 and February 2024. The study population consisted of patients with a history of prior lumbar fusion surgery, either at our institution or at outside hospitals, regardless of when the prior surgery was performed, who failed to achieve adequate symptom relief despite at least 3 months of nonoperative management. All patients completed a minimum 12-month follow-up period. All procedures were performed by two independent spine surgeons proficient in both lateral and posterior approaches, each with more than 15 years of surgical experience.

Patients were included if they had radiologically confirmed ASD cranial to a previous posterior fusion and presented with symptomatic back pain, leg pain, or neurogenic claudication that had not responded to at least 6 months of conservative management. We excluded patients with active infection or severe osteoporosis (bone mineral density T-score ≤ −2.5 with osteoporotic fracture history) that would preclude instrumentation, previous lateral surgery at the target level, or medical comorbidities that contraindicated general anesthesia.

### 2.2. Clinical and Radiological Assessment

Clinical outcomes were assessed using validated patient-reported outcome measures at preoperative baseline and at regular postoperative intervals, including 3, 6, and 12 months. The Visual Analog Scale (VAS) was used to quantify pain intensity for both back pain and leg pain—with scores ranging from 0, indicating no pain, to 10, indicating worst imaginable pain. Functional disability was evaluated using the Oswestry Disability Index (ODI), which assesses the impact of back pain on activities of daily living.

**Figure 1:**
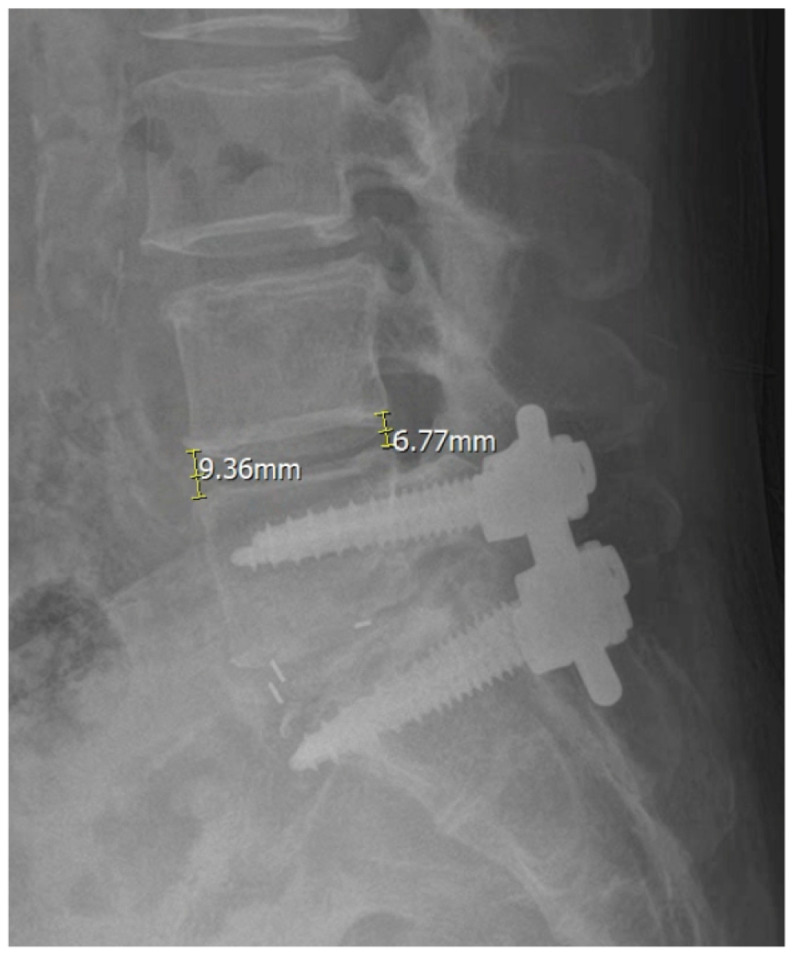
Measurement of anterior disk height (ADH) and posterior disk height (PDH) on lateral radiographs.

Comprehensive radiological evaluation was performed using standing anteroposterior and lateral radiographs obtained preoperatively and at 3 and 12 months postoperatively and at the final follow-up. We measured lumbar lordosis (LL) as the Cobb angle from L1 to S1 and segmental lordosis (SL) as the Cobb angle at the operated level. Pelvic tilt (PT) was recorded. Sagittal vertical axis (SVA) was measured as the horizontal distance from the C7 plumb line to the posterosuperior corner of S1. Disk height (DH) was measured at both anterior and posterior margins of the vertebral endplates (Figure 1). Solid fusion was defined as continuous bridging bone across the disk space with less than 3 degrees of angular motion on dynamic flexion–extension radiographs [12,13].

ADH was measured as the vertical distance between the anterior margins of the superior and inferior vertebral endplates at the operated level. PDH was measured as the corresponding vertical distance between the posterior endplate margins.

All measurements were independently performed by two board-certified neurosurgeons with more than 10 years of clinical experience. Each observer obtained two separate measurements, and the average values were used to assess both intraobserver and interobserver reliability. Reliability was evaluated using intraclass correlation coefficients (ICCs) calculated with a two-way random-effects model and absolute agreement type (Table 1).

### 2.3. Statistical Analyses

Statistical analyses were performed using SPSS Statistics, version 25.0 (IBM Corp., Armonk, NY, USA). Continuous variables are presented as mean ± standard deviation. Paired *t*-tests were used to evaluate preoperative and postoperative clinical or radiological changes. A *p*-value of less than 0.05 was considered statistically significant.

### 2.4. Surgical Technique

All procedures were performed with the patient in the prone position on a Jackson table equipped with chest and hip pads. The chest pad was positioned above the nipple line to provide adequate thoracic support while avoiding compression of respiratory excursion. Hip pads were placed below the anterior superior iliac spine, with care taken to avoid excessive caudal placement that might compress the femoral nerve. Hip and knee extension were maintained throughout the procedure to optimize the working corridor through the psoas muscle. General anesthesia was administered without neuromuscular blockade to allow intraoperative neuromonitoring.

After fluoroscopic localization of the target level, a lateral skin incision measuring approximately 3 to 4 cm was made. Sequential dilators were then used to create a working corridor through the psoas muscle under continuous neuromonitoring. Throughout the approach, triggered electromyography was employed before retractor application to confirm safe passage through the psoas muscle and minimize risk of lumbar plexus injury. Following adequate exposure, diskectomy and endplate preparation were performed, and a 3D-printed titanium interbody cage filled with bone morphogenetic protein was inserted into the disk space under fluoroscopic guidance.

After interbody cage placement, supplemental posterior instrumentation was performed through a paraspinal intermuscular approach, without patient repositioning. The existing rods were removed, and the construct was cranially extended using newly inserted pedicle screws and longer replacement rods. No surgical drains were routinely placed.

## 3. Results

### 3.1. Patient Demographics and Operative Characteristics

The study cohort comprised 30 patients with a mean age of 64.1 ± 15.1 years and a mean body mass index of 27.2 ± 4.5 kg/m^2^. The distribution of treated levels reflected typical patterns of adjacent segment degeneration, with L3–4 being the most common level in 16 patients (53.3%), followed by L4–5 in 10 patients (33.3%) and L2–3 in 4 patients (13.3%). The mean operative time for the entire procedure was 113.8 ± 26.6 min, with a mean fluoroscopic exposure of 29.5 ± 15.9 shots and a mean estimated blood loss of 105.8 ± 69.3 mL (Table 2).

### 3.2. Clinical Outcomes

All patients demonstrated improvement in pain and functional status at the 12-month follow-up. VAS scores for back pain decreased from a mean of 8.0 preoperatively to 3.3 in 12 months, representing an improvement of 4.7 ± 1.2 points (*p* < 0.05). Similarly, leg pain scores decreased from 8.6 to 2.1, showing an improvement of 6.5 ± 2.2 points (*p* < 0.05). The ODI improved by 28.5 ± 11.0 points (*p* < 0.05) (Table 3).

### 3.3. Radiological Outcomes

Radiological parameters showed favorable changes following surgery. Lumbar lordosis increased by 6.3 ± 3.9 degrees (*p* < 0.05), and segmental lordosis at the operated level increased by 5.1 ± 1.2 degrees (*p* < 0.05). Disk height restoration was achieved at all operated levels, with anterior disk height increasing by 8.1 ± 2.2 mm and posterior disk height increasing by 6.3 ± 1.2 mm. When present preoperatively, spondylolisthesis improved by a mean of 4.4 ± 3.7 mm. Sagittal vertical axis improved by 0.1 ± 2.1 mm, and pelvic tilt decreased by 0.5 ± 3.2 degrees. All operated levels exhibited solid fusion at the 12-month follow-up (Table 3).

ICC analysis demonstrated consistent reproducibility across all measured radiologic parameters, including pelvic parameters. Preoperative ICC values ranged between 0.814 and 0.942, while postoperative values ranged between 0.832 and 0.963 (Table 1).

### 3.4. Complications

Five patients (16.7%) experienced transient neurological symptoms that were resolved spontaneously without intervention. These neurological symptoms occurred in different patients, with no overlap between symptom types. Specifically, three patients (10.0%) developed transient thigh numbness, which was considered consistent with temporary psoas muscle irritation or minor lumbar plexus neuropraxia, while two patients (6.7%) experienced transient hip flexion weakness related to psoas muscle manipulation.

One case of intraoperative cage subsidence (3.3%) was identified and managed during the procedure; however, this event was not associated with postoperative neurological symptoms, clinical deterioration, instrumentation failure, or the need for revision surgery. Overall, six patients (20.0%) experienced at least one perioperative complication. Notably, all observed complications were minor in severity, and all neurological symptoms resolved spontaneously during follow-up. No major complications, including vascular injury, permanent neurological deficit, dural tear, infection, or wound complication, were observed (Table 4).

### 3.5. Illustrative Case

We present the case of an 80-year-old female who developed symptomatic cranial adjacent segment degeneration at L3–4 following posterior lumbar interbody fusion at L4–5, performed 4 years ago. Her primary complaint was progressive right anterior thigh pain. Preoperative pain assessment revealed VAS scores of 8 for back pain and 7 for leg pain. Standing radiographs showed decreased disk height at L3–4 with maintained alignment at the previously fused L4–5 segment. MRI demonstrated severe disk degeneration at L3–4 with central canal and bilateral foraminal stenosis.

**Figure 2:**
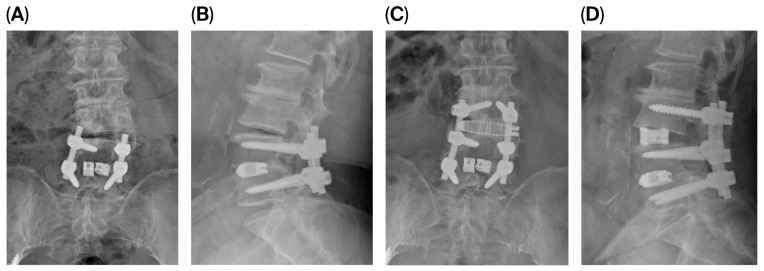
Anteroposterior (AP) and lateral radiographs before and after surgery in a representative case of prone single-position lateral lumbar interbody fusion (PSP-LLIF). (**A**,**B**) Preoperative and (**C**,**D**) 12-month postoperative AP and lateral standing radiographs of an 80-year-old female following PSP-LLIF at L3–4 with supplemental posterior instrumentation from L3 to L5. Postoperative radiographs demonstrate restored L3–4 disk height, increased lumbar lordosis (LL) and segmental lordosis (SL), and improvement in anterior disk height (ADH) and posterior disk height (PDH), with confirmed fusion and no evidence of hardware loosening, cage subsidence, or migration at 12 months.

The patient underwent PSP-LLIF at L3–4 with supplemental posterior instrumentation extending from L3 to L5, incorporating the L3–4 level into the existing fusion construct. The entire procedure was completed in 110 min without position change. Intraoperative fluoroscopy confirmed appropriate cage positioning. At 12-month follow-up, VAS scores improved to 3 for back pain and 2 for leg pain. Radiological measurements showed LL increased from 27.87 degrees to 36.94 degrees, SL increased from 6.16 degrees to 15.99 degrees, ADH increased from 7.03 mm to 12.87 mm, and PDH increased from 5.85 to 9.39 (Figure 2). Dynamic radiographs confirmed solid fusion without evidence of hardware loosening, cage subsidence, or migration.

## 4. Discussion

### 4.1. Rationale for Single-Position Surgery and Its Clinical Significance

The concept of single-position spine surgery emerged approximately a decade ago to address the time and risk associated with intraoperative repositioning. A systematic review and meta-analysis by Mill et al. provided valuable benchmarking data by comparing outcomes between single-position and traditional repositioned approaches [14]. Their analysis included 942 patients in the single-position group versus 254 in the repositioned group and demonstrated that single-position surgery reduced mean operative time from 188.7 min to 127.5 min (*p* < 0.001), representing approximately 60 min of time savings. Additionally, single-position approaches shortened hospital length of stay from 6.6 days to 2.9 days (*p* < 0.001) while maintaining equivalent clinical outcomes. Our mean operative time of 113.8 min is consistent with these findings and confirms the efficiency of eliminating position change (Table 2).

### 4.2. Advantages of the Prone Single-Position Approach

While both prone and lateral single-position approaches eliminate repositioning, several factors favor the prone approach. Performing posterior screw fixation in the lateral decubitus position requires surgeons to develop new skills outside their standard training, and the technical difficulty increases substantially with longer constructs or extension of existing fusions [15]. In contrast, the prone position allows surgeons to perform posterior instrumentation using familiar techniques and ergonomics. Furthermore, the prone position naturally facilitates lordosis restoration through hip extension and gravitational effects on the abdominal contents, whereas the lateral position provides no such biomechanical advantage [16,17]. Our results support this theoretical benefit, with lumbar lordosis increasing by 6.3 degrees and segmental lordosis by 5.1 degrees. Finally, the prone position allows for additional posterior decompression procedures, such as laminectomy or foraminotomy, if needed, which are technically challenging or impossible to perform in the lateral position.

### 4.3. Complication Profile in the Context of the Existing Literature

Among the complications identified in the present study, a total of five cases of transient hip weakness and thigh numbness were observed. The primary mechanism underlying these complications has been suggested to be lumbar plexus irritation and psoas muscle manipulation [18,19,20]. Previous studies of conventional lateral lumbar interbody fusion (LLIF) have reported a favorable natural course of these neurologic complications. For example, hip flexion weakness decreases from approximately 13–31% in the early postoperative period to less than 2% by 6–12 months, while early postoperative thigh numbness has been reported in approximately 5–25% of patients but declines to below 1–3% within 6–12 months [18,21]. In contrast, data specifically addressing the time-dependent recovery of neurologic complications following PSP-LLIF are still lacking. Meanwhile, although differences in patient positioning between LLIF and PSP-LLIF may influence the relative position of the psoas muscle and lumbar plexus, an earlier analysis reported that the overall neurological complication profile of PSP-LLIF was comparable to that of LLIF [22].

A comprehensive systematic review published in 2023 analyzed complications from 10 studies, encompassing 286 patients, who underwent PSP-LLIF [23]. Specific postoperative complications included transient thigh or groin symptoms in 13.3% of patients and hip flexor weakness in 17.8%, while intraoperative subsidence occurred in 3.8%. Our observed rates of transient thigh numbness (10%), hip flexion weakness (6.7%), and intraoperative subsidence (3.3%) fall within or below these published ranges. More importantly, we observed no vascular injuries or dural tears. The absence of dural tears is particularly noteworthy, given that we were operating in a revision setting where posterior approaches carry dural tear rates of 13.2% to 21.4%.

### 4.4. Anatomical Implications of Prone Positioning

In the prone position, gravity causes anterior and inferior displacement of the abdominal contents [24,25]. Compared to the lateral decubitus position, the position of the psoas muscle moves posteriorly relative to the vertebral body [17,25]. The clinical significance lies in how these changes affect the position of neurovascular structures, particularly the femoral nerve and lumbar plexus.

Comparative analysis demonstrates that the femoral nerve undergoes approximately 4 mm of posterior displacement in the prone position compared to lateral positioning. Specifically, the nerve location moves from 12 mm out of 29 mm (41% of the anteroposterior disk space dimension) in the lateral position to 8 mm out of 27 mm (30%) in the prone position. This posterior displacement theoretically expands the safe working zone anterior to the nerve, potentially reducing the risk of nerve injury during the transpsoas approach [26]. However, this anatomical advantage is counterbalanced by gravity-associated ventral retractor drift, which can bias surgical instruments toward the great vessels and the anterior longitudinal ligament [27,28].

### 4.5. Technical Considerations for Safe Execution

Successful PSP-LLIF requires attention to several technical details. Patient positioning must be precise, with the chest pad placed above the nipple line and the hip pads positioned below the anterior superior iliac spine but not excessively caudal so as to avoid femoral nerve compression. The contralateral hip bolster provided stable counter-pressure during trial and cage insertion, particularly important when mallet impaction was required, preventing patient movement that could compromise surgical accuracy. This stabilization was especially critical when approaching L4–5 in patients with high iliac crests. We have found that tilting the patient approximately 15 degrees toward the contralateral side improves surgeon ergonomics while maintaining the ability to obtain perpendicular fluoroscopic imaging.

The most critical technical challenge relates to gravitational effects on instruments and cage trajectory. In the prone position, gravity exerts an anterior pull on all instruments, increasing the tendency for anterior cage placement and risking injury to the anterior longitudinal ligament or great vessels. We address this through three main strategies. First, we maintain strict perpendicular C-arm alignment throughout the procedure, as even slight angulation can result in progressive anterior drift of the working trajectory. Second, we place the skin incision slightly more posteriorly than in standard lateral lumbar interbody fusion, approximately at the posterior margin of the disk space rather than the center. Third, we maintain constant awareness of this anterior tendency during diskectomy and cage insertion.

Another important technical consideration in PSP-LLIF is the risk of intraoperative cage subsidence, or endplate injury, during diskectomy and cage insertion. To the best of our knowledge, there are currently no standardized or systematic studies addressing the prevention or management of intraoperative cage subsidence specifically in PSP-LLIF. In our practice, when intraoperative endplate injury is evident, the cage position is adjusted anteriorly or posteriorly under fluoroscopic guidance based on the location of the endplate injury. In addition, to prevent further endplate damage, the initially inserted cage is exchanged for one with a lower height and a wider footprint. Finally, posterior instrumentation is applied with minimal compression to avoid additional cage subsidence.

Lastly, intraoperative neuromonitoring with triggered electromyography before retractor docking is mandatory. The posteriorly displaced and stretched femoral nerve may be under increased tension and potentially more vulnerable to traction injury if encountered during retractor placement. Real-time monitoring allows for immediate recognition of nerve irritation and prompt corrective action.

### 4.6. Variations in Surgical Technique: Prone Transpsoas vs. Prone Pre-Psoas Corridor

A recent technical report described an alternative prone single-position technique using the prepsoas corridor for oblique lumbar interbody fusion [29]. This approach accesses the disk space anterior to the psoas muscle, theoretically avoiding direct muscle transgression and reducing the risk of lumbar plexus injury. The prepsoas approach may result in lower rates of postoperative hip flexor weakness or thigh numbness compared to transpsoas techniques. However, the prepsoas corridor brings the working trajectory closer to the great vessels and sympathetic plexus, requiring meticulous attention to vascular anatomy.

The choice between prone transpsoas and prone prepsoas approaches depends on multiple factors, including surgeon experience, patient anatomy, and target level characteristics. At L4–5 and L5–S1, the prepsoas corridor may be more accessible due to the lateral position of the psoas muscle at these levels. At L2–3 and L3–4, both corridors are generally feasible. The transpsoas approach provides more direct access to the posterior disk space and may be advantageous when posterior decompression through aggressive diskectomy is desired. The prepsoas approach offers a safer neurological profile at the expense of increased vascular considerations. Both techniques share the fundamental innovation of eliminating intraoperative repositioning while maintaining the benefits of lateral interbody fusion.

### 4.7. Study Limitations

This study has several limitations. The retrospective design and relatively small sample size of 30 patients limit statistical power for detecting uncommon complications. The study cohort represents all consecutive patients treated for symptomatic cranial ASD using PSP-LLIF. The 12-month follow-up period, while adequate for assessing early fusion and short-term clinical outcomes, is insufficient to fully evaluate long-term outcomes such as recurrent ASD, fusion durability, instrumentation loosening, or reoperation rates. Accordingly, the present findings should be interpreted as early clinical and radiological results rather than definitive long-term evidence. The absence of a control group precludes direct statistical comparison with traditional lateral positioning or posterior approaches. Outcomes may be influenced by surgeon experience and learning curve effects, potentially limiting generalizability to other institutions or less experienced surgeons. Finally, as the study population was limited to patients with cranial ASD, the results may not apply to other indications, such as primary degenerative disease or deformity correction.

### 4.8. Clinical Implications and Future Directions

Our results demonstrate that PSP-LLIF effectively treats symptomatic cranial ASD. The technique achieved a mean operative time of 113.8 min, with minimal blood loss of 105.8 mL. Clinical outcomes were excellent, with VAS improvements of 4.7 points for back pain and 6.5 points for leg pain, as well as an ODI improvement of 28.5 points. Radiological outcomes showed successful lordosis restoration with increases of 6.3 degrees in LL and 5.1 degrees in SL. The transient neurological symptom rate of 16.7% was acceptable and self-limited, with all symptoms resolving without intervention.

For surgeons considering this technique, the critical factors for success include understanding the anatomical changes specific to prone positioning, maintaining perpendicular fluoroscopic alignment to prevent anterior drift, employing comprehensive intraoperative neuromonitoring, and recognizing the tendency toward anterior cage placement while preventing excessive anterior positioning that might risk vascular injury.

Future research should include direct comparisons between prone transpsoas and prepsoas approaches to define optimal indications for each technique. Longer follow-up studies are needed to assess outcome durability and the rates of subsequent adjacent segment degeneration. Cost-effectiveness analyses would provide valuable information for healthcare systems. Finally, refinement of patient selection criteria will help identify which patients benefit most from prone single-position techniques versus traditional approaches.

## 5. Conclusions

PSP-LLIF is an effective and safe option for managing symptomatic cranial ASD, providing significant improvements in pain, function, and sagittal alignment without the need for intraoperative repositioning. In our series, no vascular injury, dural tear, or permanent neurological deficit occurred, and transient neurological symptoms, although not uncommon, resolved spontaneously. With proper attention to prone-position anatomy and neuromonitoring, PSP-LLIF can serve as a reliable alternative to revision posterior surgery.

## Figures and Tables

**Table 1:** Inter- and intraobserver reliability of radiological measurements.

	Pre		Post	
	Intraobserver	Interobserver	Intraobserver	Interobserver
SVA	0.887	0.814	0.908	0.832
PT	0.901	0.846	0.926	0.871
LL	0.918	0.874	0.94	0.892
SL	0.876	0.823	0.903	0.845
Spondylolisthesis at the index level	0.942	0.894	0.963	0.917
ADH	0.913	0.862	0.934	0.884
PDH	0.938	0.901	0.958	0.925

SVA, sagittal vertical axis; PT, pelvic tilt; LL, lumbar lordosis; SL, segmental lordosis; ADH, anterior disk height; PDH, posterior disk height.

**Table 2:** Details of patients with PSP-LLIF.

	Variables
Numbers of patients	30
Age (years)	64.1 ± 15.1
BMI (kg/m^2^)	27.2 ± 4.5
Levels	
L2–3	4
L3–4	16
L4–5	10
Operation time	113.8 ± 26.6
X-ray exposure	29.5 ± 15.9 shots
EBL	105.8 ± 69.3

PSP-LLIF, prone single-position lateral lumbar interbody fusion; EBL, estimated blood loss.

**Table 3:** Pre- and postoperative clinical and radiological outcomes.

	Preop	Postop (12 Mos)	*p*-Value	Δ
VAS (back)	8.0 ± 2.3	3.3 ± 1.7	<0.001 *	4.7 ± 1.2
VAS (leg)	8.6 ± 2.0	2.1 ± 2.4	<0.001 *	6.5 ± 2.2
ODI	39.0 ± 9.8	10.5 ± 10.2	<0.001 *	28.5 ± 11.0
SVA (cm)	3.6 ± 4.5	3.5 ± 4.3	0.84	0.1 ± 2.1
PT (°)	21.8 ± 7.4	21.3 ± 7.0	0.41	0.5 ± 3.2
LL (°)	37.8 ± 9.6	44.1 ± 5.7	<0.001 *	6.3 ± 3.9
SL (°)	11.2 ± 6.4	16.3 ± 5.2	<0.001 *	5.1 ± 1.2
Spondylolisthesis at the index level (mm)	6.8 ± 3.4	2.4 ± 2.8	<0.001 *	4.4 ± 3.7
ADH (mm)	5.1 ± 2.2	13.2 ± 2.6	<0.001 *	8.1 ± 2.2
PDH (mm)	4.7 ± 2.0	11.0 ± 2.3	<0.001 *	6.3 ± 1.2

VAS, visual analog scale; ODI, Oswestry Disability Index; SVA, sagittal vertical axis; PT, pelvic tilt; LL, lumbar lordosis; SL, segmental lordosis; ADH, anterior disk height; PDH, posterior disk height. * *p* < 0.05, statistically significant.

**Table 4:** Postoperative complications.

Complication Type	Severity	No. of Patients (%)	Progress
Transient neurological symptoms	Minor	5 (16.7%)	-
- Thigh numbness		3 (10.0%)	Recovered without intervention (immediate or POD 1 onset; improved by 3 months)
- Hip flexion weakness		2 (6.7%)	Fully resolved (immediate onset; POD 6 and POD 18)
Intraoperative cage subsidence	Minor	1 (3.3%)	Managed intraoperatively
Vascular injury	Major	0	-
Permanent neurological deficit	Major	0	-
Dural tear	Major	0	-
Infection or wound complication	Major	0	-
Total complication rate		6 (20.0%)	

POD, postoperative day.

## Data Availability

The datasets generated and analyzed during the current study are available from the corresponding author on reasonable request, subject to institutional review board approval and patient privacy protections.
